# 4DCT and CBCT based PTV margin in Stereotactic Body Radiotherapy(SBRT) of non-small cell lung tumor adhered to chest wall or diaphragm

**DOI:** 10.1186/s13014-016-0724-5

**Published:** 2016-11-15

**Authors:** Yi Li, Jing-lu Ma, Xin Chen, Feng-wen Tang, Xiao-zhi Zhang

**Affiliations:** Department of Radiation Oncology, The First Affiliated Hospital, Xi’an Jiaotong University of Medical College, Xi’an, Shaanxi 710061 China

**Keywords:** 4DCT, CBCT, SBRT, PTV margin, NSCLC

## Abstract

**Background:**

Large tumor motion often leads to larger treatment volumes, especially the lung tumor located in lower lobe and adhered to chest wall or diaphragm. The purpose of this work is to investigate the impacts of planning target volume (PTV) margin on Stereotactic Body Radiotherapy (SBRT) in non-small cell lung cancer (NSCLC).

**Methods:**

Subjects include 20 patients with the lung tumor located in lower lobe and adhered to chest wall or diaphragm who underwent SBRT. Four-dimensional computed tomography (4DCT) were acquired at simulation to evaluate the tumor intra-fractional centroid and boundary changes, and Cone-beam Computer Tomography (CBCT) were acquired during each treatment to evaluate the tumor inter-fractional set-up displacement. The margin to compensate for tumor variations uncertainties was calculated with various margin calculated recipes published in the exiting literatures.

**Results:**

The means (±standard deviation) of tumor centroid changes were 0.16 (±0.13) cm, 0.22 (±0.15) cm, and 1.37 (±0.81) cm in RL, AP, and SI directions, respectively. The means (±standard deviation) of tumor edge changes were 0.21 (±0.18) cm, 0.50 (±0.23) cm, and 0.19 (±0.44) cm in RL, AP, and SI directions, respectively. The means (±standard deviation) of tumor set-up displacement were 0.03 (±0.24) cm, 0.02 (±0.26) cm, and 0.02 (±0.43) cm in RL, AP, and SI directions, respectively. The PTV margin to compensate for lung cancer tumor variations uncertainties were 0.88, 0.98 and 2.68 cm in RL, AP and SI directions, which were maximal among all margin recipes.

**Conclusions:**

4DCT and CBCT imaging are appropriate to account for the tumor intra-fractional centroid, boundary variations and inter-fractional set-up displacement. The PTV margin to compensate for lung cancer tumor variations uncertainties can be obtained. Our results show that a conventional 1.0 cm margin in the SI plane dose not suffice to compensate the geometrical variety of the tumor located in lower lobe and adhered to chest wall and diaphragm.

## Background

Lung cancer is one of the leading causes of death in cancer patients in China. According to the literature, 3-year local control rate of the lung cancer is only 66% in conventional conformal radiotherapy [1]. The stereotactic body radiation therapy (SBRT) is a new technology which can effectively improve the treatment effect of lung cancer. A meta-analysis of 34 lung cancer SBRT studies including 2578 patients reported that 3-year local control rate for tumors measuring greater than 3 cm was 87% [2]. SBRT delivers high dose in fewer fractions (3–10 fractions) than conventional radiotherapy which may increase toxicity in adjacent organs at risk (OAR). It is therefore critical to minimize the irradiated volume of healthy tissue when delivering the prescribed dose to tumor in SBRT.

However, a variety of geometrical uncertainty such as respiratory motion [3, 4], baseline variation [5–8] and set-up error [9], limit the precision of radiation therapy (RT) for lung cancer. According to ICRU report 62#, internal margin (IM) and set-up margin (SM) should be included in the PTV to compensate geometrical uncertainties including tumor centroid movement [10], tumor boundary [11, 12] and set-up displacement, especially in SBRT. However, the tumor boundary displacement merely discussed about margin calculation previously. It would be assessed in present study.

As a standard modality to compensate tumor motion, four-dimensional CT (4DCT) has been generally applied to the calculation of the extent margin of tumor motion [13], and is also incorporated into treatment planning. When only areas with tumor appearance are included in the target volume definition, there is a significant reduction of the mean volume by the use of 4DCT [14]. Generally, the need of contour gross tumor volume (GTV) in up to ten phases of respiration is a time-consuming procedure for the routine clinical use of 4DCT scans. To reduce the workload of multiple contour delineations in 4DCT, maximum intensity projection (MIP) and averaged intensity (AI) of 4DCT image are reconstructed in order to be used in the treatment planning, MIP provide a 3D CT scan whose vowels’ values are the greatest vowels’ intensity values throughout the 4D CT dataset. AI provides a 3D CT scan whose vowels’ values are the arithmetic mean of the 4D CT scans [15]. However, the tumor contoured with the MIP or AI series cannot totally represent the true tumor size, location and shape, especially adhered to chest wall or diaphragm [15]. To correctly assess the tumor motion and tumor shape, ten phase of the 4DCT were contoured in our study.

Tumor localization is also a significant issue in NSCLC SBRT, the major focus has been the development of image-guidance system. Several image guidance techniques such as electronic portal imaging device (EPID) [16] and CBCT [17], have been adapted to narrow the set-up margin. Among them, three-dimensional (3D) matching, coupled with CBCT provides results that have greater accuracy than two-dimensional matching, coupled with EPID [18]. Generally, with the repeating CBCT imaging, intra-fractional and inter-fractional set-up displacement can be assessed for the lung cancer patients’ set-up displacement using KV X-ray,such as fluoroscopy, CBCT and four dimensional CBCT (4D-CBCT) [19–21]. CBCT imaging is sufficient to account for the inter-fractional tumor variation and setup variation based on the bone structure, which was adopted in our study. Furthermore, size, patients characteristics are also significant factors to influent patients’ set-up margin [22], but these factors are negligible in the set-up margin.

The present study would evaluate changes in tumor motion magnitude and set-up error by 4DCT at planning and CBCT at treatment, and calculated the clinical target volume (CTV)-planning target volume (PTV) margins to compensate for these changes, which may minimize the risk of complications and tumor failures. The aim of the present study is to decide whether conventional 1.0 cm margin in the SI plane and 0.5 cm margin in the other plane suffice to compensate the geometrical variety of the tumor adhere to chest wall and diaphragm.

## Methods

### Patient and tumor characteristics

Between February 2013 and June 2015, 20 patients were recruited to this study to assess PTV margin. Table 1 summarizes the patient and tumor’s general information and detailed characteristics.

**Table 1:** Patients and tumor generally characteristics of 20 patients in this study

	Number
Sex	
Male	12
Female	8
Age (years)	
> 60	7
≤ 60	13
Tumor volume (cm^3^)	
> 40	9
≤ 40	11
Side	
Right	6
Left	14
Site	
Chest wall adherent	15
Diaphragm adherent	5

The studies were approved by the Institutional Committee for Clinical Research and the Local Ethics Committee in The First Affiliated Hospital, Xi’an Jiaotong University of medical college. Eligible patients were given written informed consent.

### 4DCT simulation and image acquisition

During the CT simulation, all patients were immobilized using thermoplastic mask in the supine position with arms raised above the head. Then, each patient received a helical treatment planning three-dimensional computed tomography (TP-3DCT) and an additional 4DCT simulation under free breathing condition on a 16-slice CT scanner (Big bore, Philips Medical Systems, Cleveland, OH). For TP-3DCT, each scan’s rotation time took about 1 s to acquire image dates. The other parameters were as follows: pitch 0.85, 120KV and 400mAs. During 4DCT scanning, the respiratory signal was acquired by a pressure sensor (“pneumonia bellows”). The pressure in the bellows decreases as the patient inhales, with the opposite happening as the patient exhales. Then, the pressure change was measured by a remote pressure sensor which provided a direct-current voltage to an analog-to-digital converter. 4DCT images were reconstructed and then sorted into 10 respiratory phases, with 0% representing end-inhalation and 50% representing end-exhalation. Prospectively phase-binned image sets was been used to extract displacement binned 4DCT. Both the TP-3DCT and 4DCT images were reconstructed with a thickness of 3 mm and then transferred to a Pinnacle V. 9.0y treatment planning system (Philips Medical Systems, Fitchburg, USA).

### Manual contouring and treatment planning

**Fig. 1:**
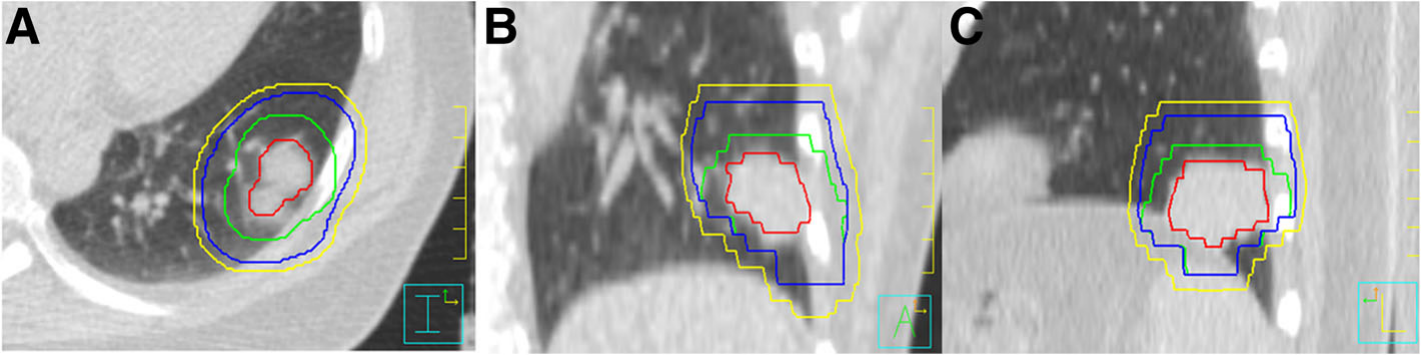
Phase 0% of four-dimensional computed tomography (4DCT) image for an protocol NSCLC patient. (**a**) transverse (**b**) sagittal (**c**) coronal; Gross tumor volume (GTV) of phase 0% in *Red*; Clinical target volume (CTV) of phase 0% in *Green*; Internal clinical target volume (ITV) in *blue*; Planning target volume (PTV) in *Yellow*

For target volume definition, the 4DCT phases display the extreme of tumor positions for each spatial direction. After the matching of CT-cubes between 4DCT phases and 3DCT phase in the Philips treatment planning system, GTV contours were manually delineated on the 10 phases of the 4DCT scan using the lung window setting by the same physician to make the inter-observer lowest as soon as possible (L = −600Hu, W = 1600Hu [20]), and CTV_4D_ was defined as GTV_4D_ plus an isotropic margin of 0.6 cm for adenoid carcinoma and the one of 0.8 cm for squamous carcinoma. The combined volume of each CTVs in 10 phases was defined as internal CTV_4D_ (ITV). The modification to form the PTV_4D_ was performed by adding an isotropic margin of 3 mm to ITV to account for set-up inaccuracies in all direction (Fig. 1). The PTV_4D_ contours on 4DCT phases were projected to the contour named PTV on the TP-3DCT.

For treatment planning, every patient received an individual treatment plan and the plan calculation were performed on 3DCT, 10 patients were treated with the IMRT technology, and the others with conformal arc technology. All patients were prescribed total doses of 48Gy in 4 fractions [16, 23, 24] with the 95% isodose covering the PTV.

### Tumor boundary change definition

**Fig. 2:**
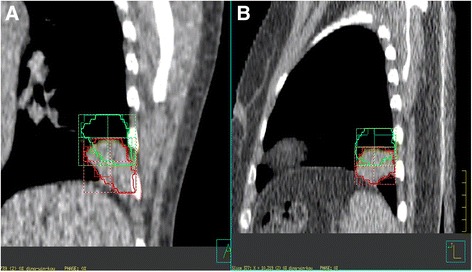
Phase 0% of four-dimensional computed tomography (4DCT) image for an protocol NSCLC patient. (**a**) coronal (**b**) sagittal; Gross tumor volume (GTV) of phase 0 % (GTV_0%_) in *Red*; Gross target volume (GTV) of phase 50 % (GTV_50%_) in *Green*

Figure 2 shows tumor boundary change definition. Ten beams with gantry angle 0° and 90° were used and conformed to GTV of each phase with centroid of corresponding GTV for each patient. The values of jaw location in the RL, and SI directions with beam of angle 0° were collected. Then the same values with beam of angle 90° were also collected. The variation of these values represent the boundary displacement in the RL, AP and SI direction.

### CBCT simulation and image acquisition

**Fig. 3:**
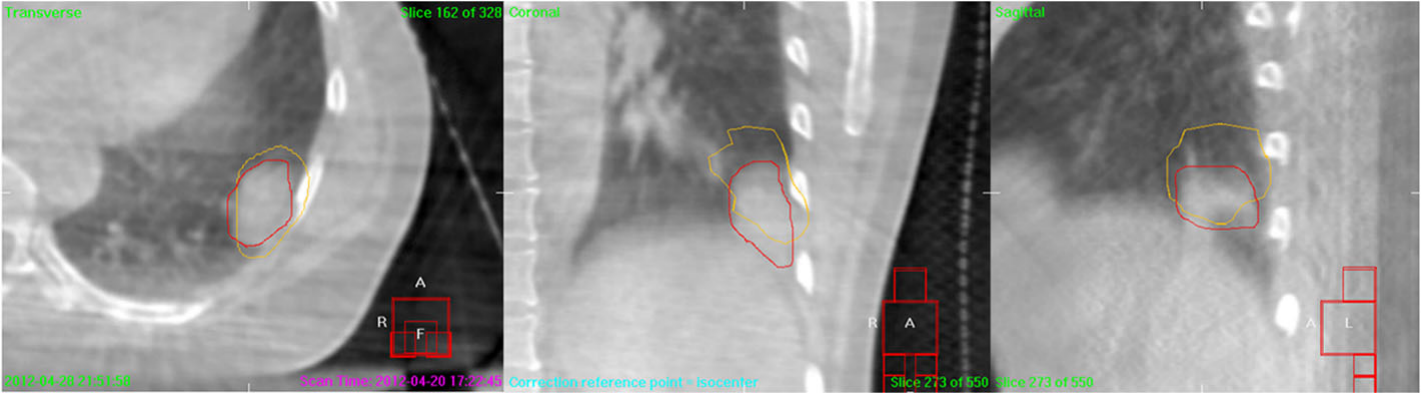
Precorrection cone-beam CT (CBCT) image for an protocol NSCLC patient in the thermoplastic mask. TP-3DCT GTV in *Yellow*; Precorrection GTV in *Red*

The on-board imager integrated in a Synergy medical linear accelerator (Elekta, Stockholm, Sweden) was adopted to acquire CBCT images. Before acquiring the image data, each patient was immobilized in the same position as that used in 4DCT scan. A 120 kV x-ray tube voltage and 400mAs current were used for generating middle-resolution images. The scan angle was from −178° to 178°. All CBCT images were reconstructed with a thickness of 3 mm. After each patient were initial positioned, a CBCT was acquired and registered to the planning TP-3DCT based on the location of vertebral bodies (bony landmarks) (Fig. 3). The target position error was corrected by shifting the treatment couch. If the position error was ≤2 mm in all three direction, the patient was treated without shifting couch; else couch was shifted and a second CBCT was acquired to measure the residual error until a residual error was ≤2 mm [19]. At the end of treatment, a CBCT scan was finally performed to assess the intra-fractional set-up displacement.

### Respiration motion analysis

Centroid position and boundary of the respective CTV_4D_ were calculated and compared. The centroid shift of respective CTV_4D_ was represented as tumor position variation, and the tumor boundary change of respective CTV_4D_ was represented as tumor shape variation during respiration. The 3D spatial motion vector of the individually CTV_4D_ centroid and boundary variation were evaluated according to the recipe as follows [25]

$$ V=\sqrt{RL^2+{AP}^2+{SI}^2}. $$

### Margin calculation

Existing literature has proposed approaches to calculate the PTV margin based on systematic and random uncertainties (see Table 2) [8, 26–28], which were applied for the PTV margin calculation in our study. Among these approaches, the recipes approached by Stroom and van Herk are widely adopted in current clinical treatment. In all recipes, ∑ represents the standard deviation (SD) of systematic errors and *σ* is the root-mean square of random errors. In this study, the systematic errors include the standard deviat55ion (SD) of the centroid movement denoted as ∑_*centroid*_, the tumor boundary changes denoted as ∑_*boundary*_ and set-up displacement denoted as ∑_*setup*_ of each patient. As each patient had one 4DCT scan, the random errors of the centroid and boundary changes were zero, and the total random errors equal to set-up random errors. As a result, the total SD of systematic errors ∑_*total*_ and random errors were evaluated with the recipe: ${\sum}_{total}=\sqrt{\sum_{centroid}^2+{\sum}_{boundary}^2+{\sum}_{setup}^2}$, *σ*_*total*_ = *σ*_*setup*_.

**Table 2:** Summary of various published recommendations for margins around target volumes (CTV)

Author	Region	Recipe
Stroom et al.(1999a) [26]	PTV	2∑ + 0.7*σ*
Van Herk et al.(2000) [27]	PTV	2.5∑ + 0.7*σ*
Parker et al. (2002) [28]	PTV	$\sum +\sqrt{\left({\sigma}^2+{\sum}^2\right)}$
Snoke JJ (2007)	PTV	2.5∑ + *β*(*σ* ^2^ + *σ*_*P*_^2^)^1/2^ − *βσ*_*P*_

Symbols: ∑, standard deviation of systematic uncertainties; σ, standard deviation of statistical (random) uncertainties. *σ*_*P*_ =0.64, *β* =0.84.

### Data analysis

SPSS 19.0 was used for statistical analysis. Differences were considered significant for *P* <0.05. For test of correlation, it is Person’s rank test which was used.

## Results

### Tumor centroid and boundary changes

**Fig. 4:**
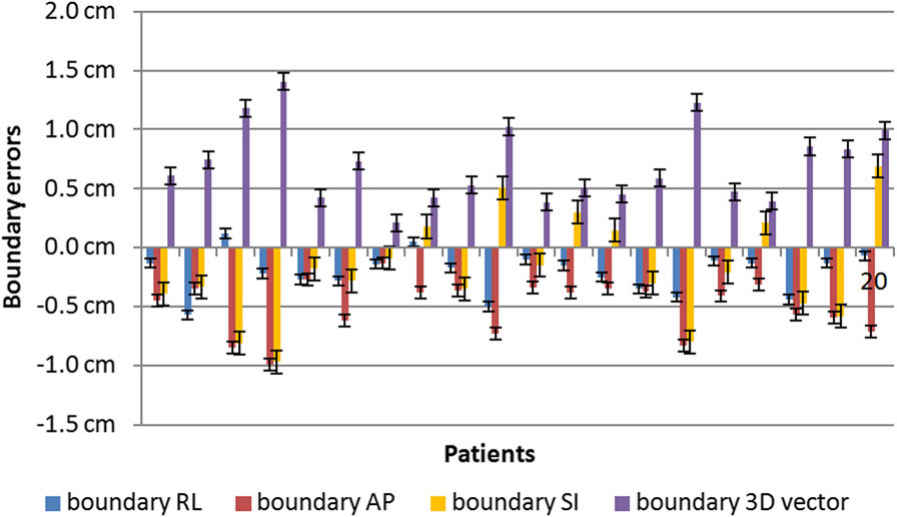
Boundary errors in RL,AP,and SI directions and 3D vector for 20 patients

**Fig. 5:**
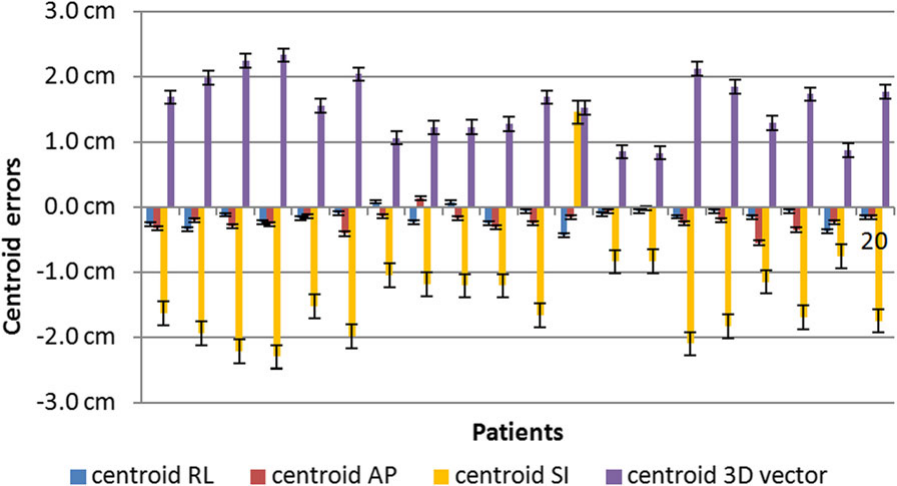
Centroid position errors in RL,AP,and SI directions and 3D vector for 20 patients

Figures 4 and 5 show the tumor boundary and centroid changes for 20 patients. A positive value in the right-left (RL), anterior-posterior (AP), and superior-inferior (SI) directions represents a shift in the right, anterior, and inferior directions, respectively. We did not find any statistically significant correlation between tumor centroid displacement or boundary changes and patients’ age. The mean 3D centroid change value was 1.55 ± 0.46 cm, with a maximum at 2.33 cm. Tumor centroid had a greater mean changes in SI direction than in other direction (SI vs RL: t = 0.587,P < 0.05; SI vs AP: t = 0.547, P < 0.05). The mean 3D boundary change value was 0.70 ± 0.33 cm, with a maximum at 1.40 cm. Tumor boundary motion had a greater mean displacement in AP direction than in other directions, but it is not statistically significant, which is similar to the one observed by Gauthier et al. [12]. In all direction, the average boundary changes directions were toward to the centroid changes directions, which means the tumor shrank as expected.

### Tumor set-up errors

**Fig. 6:**
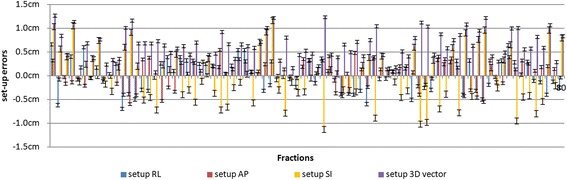
Set-up errors in in RL, AP, and SI directions and 3D vector for 80 fractions. Data were derived based on bony landmark alignments

Figure 6 shows tumor set-up errors in RL, AP, and SI directions for 80 pre-correction CBCT fractions. A positive value in the right-left (RL), anterior-posterior (AP), and superior-inferior (SI) directions represents a shift in the right, anterior, and inferior directions, respectively. The maximum set-up error were as large as 0.66 cm in RL direction, 0.54 cm in AP direction, and 1.16 cm in SI direction. The average setup errors are 0.13 ± 3.15 cm,0.37 ± 2.76 cm and 0.20 ± 5.73 cm in RL, AP and SI directions, respectively. From the distribution, we could calculated the probability of having set-up errors lager than 5 mm (which is the margin used for set-up margin) and found it to be 15.0% in RL, 6.3% in AP, and 38.8% in SI directions, respectively. The mean set-up errors had a greater value in SI direction than in other directions. (SI vs AP: r = −0.609, p <0.05; SI vs RL: r = −0.480, p > 0.05).

### PTV margin

Table 3 shows the SDs of systematic (∑) and random (*σ*) errors. One patient had one 4DCT scan at tumor CT simulation, and therefore the random errors were zero for tumor centroid and boundary displacement. Based on the SDs of set-up systematic and random errors in previous table, the pre-correction set-up margin was calculated and showed in Table 4. Among all recipes, the maximum were 0.67, 0.69 and 1.21 cm in RL, AP and SI directions, which were calculated with the recipe by van Herk et al. To compensate all geometrical uncertain motioned above, the total margin were calculated and showed in Table 4. Among all recipes, the maximum were 0.88, 0.98 and 2.68 cm in RL, AP and SI directions, which were also calculated with the recipe by van Herk et al.

**Table 3:** The Standard deviation of systematic (∑) and random (*σ*) errors

	∑			*σ*		
	RL	AP	SI	RL	AP	SI
Centroid point	0.14	0.18	0.55			
Boundary	0.19	0.21	0.17			
Set-up	0.24	0.26	0.43	0.10	0.08	0.20

Symbols: ∑, standard deviation of systematic uncertainties; σ, standard deviation of statistical (random) uncertainties

**Table 4:** CTV margin changes of NSCLC patients

Author	Recipe	Margin through set-up errors			Margin through all variation		
		ML	AP	SI	ML	AP	SI
Stroom et al. (1999a) [26]	2∑ + 0.7*σ*	0.55	0.57	0.99	0.72	0.79	2.18
van Herk et al. (2000) [27]	2.5∑ + 0.7*σ*	0.67	0.69	1.21	0.88	0.98	2.68
Parker et al. (2002) [28]	$\sum +\sqrt{\left({\sigma}^2+{\sum}^2\right)}$	0.50	0.52	0.90	0.66	0.75	2.06
Snoke JJ (2007)	2.5∑ + *β*(*σ* ^2^ + *σ*_*P*_^2^)^1/2^ − *βσ*_*P*_	0.60	0.64	1.09	0.82	0.93	2.57

## Discussion

It is reported [29] that the tumor movement in lung is not related to patient height, weight, cancer stage and lung function, and therefore these issues were not included in this study. We evaluated changes in tumor motion magnitude and set-up error by 4DCT scan at planning and CBCT scan at treatment and calculated the margins to compensate for these changes [16, 17]. To our knowledge, this is the first study to report the evaluation of tumor centroid, boundary changes and set-up displacement, which are account into the margins calculation.

We admit that at the time we performed this study, we did not perform Post-correction and post-treatment CBCT to verify that set-up displacement and tumor locatioin had not changed. However, [30, 31] existing literatures have shown that post-correction margins based on bony structure are negligible to account into PTV margin, which are less than 2 mm in three directions [24]. Other literature has shown that inter-fractional tumor position and breathing motion based on bony structure during treatment are stable. Therefore, the post-treatment margins are also negligible to account into PTV margin. In addition, we did perform post-correction and post-treatment CBCT on other patients and found that the tumor with post-correction and post-treatment displacement changed is negligible, and therefore the data have not yet been published.

The variation in the centroid changes between ITV based from 4DCT and CTV based from 3DCT was not significant [32]. Therefore, the intra-fractional tumor motion on 4DCT different phase can represent the one on the TP-3DCT. Intra-fractional tumor motion in present study was divided into tumor centroid and boundary displacement under free-breathing conditions. Centroid and boundary variation were collected starting from 0% phase to ending at a phase of maximum variation, which was 50% phase mostly. So the variation of centroid and boundary were collected starting from inferior direction to ending at superior direction. In my study, A positive value in superior-inferior (SI) directions represents a shift in the inferior directions. So the values for centroid an boundary in the graphs are negative values. Mori, S et al. [33] quantified the magnitude of intra-fraction lung tumor motion under free-breathing conditions using 4DCT and reported the mean (±standard deviation) tumor centroid motion were 0.19 (±0.16), 0.40 (±0.23), and 1.03 (±0.71)mm in the RL, AP, and SI directions respectively, which was similar with the present study result. Only the motion in SI direction was less than present study. We presume that only a few lung tumors were located in the lower lobe on previous study, resulting in their small centroid changes in SI direction.

Tumor boundary motion, which was merely discussed on previous PTV margin study, were quantified in present study and found that the maximum were 0.57, 0.99, and 0.97 cm in RL, AP and SI direction respectively. We could calculated the probability of having boundary motion lager than 5 mm and found it to be 10.0% in RL, 40.0% in AP, and 7.5% in SI directions, respectively. We presume that the tumor located in lower lobe patients was adhered to the diaphragm, resulting in their significant boundary changes. We believe that the boundary changes in intra-fractional variation is significant, especially in SBRT treatment.

Set-up error in the present study was defined as the shift from TP-3DCT to CBCT. This error includes the inter-fractional set-up error based on bony structure. The standard deviation of set-up systematic errors (∑) are 0.24, 0.26 and 0.43 cm in RL, AP and SI directions. The result was similar with the one observed by Worm, E.S. et al., which was 0.23, 0.16 and 0.45 cm [31]. The standard deviation of set-up random errors (∑) are 0.10, 0.08 and 0.20 cm in RL, AP and SI directions. The result was also similar with the one observed by Li, W ea al, which was 0.12, 0.13 and 0.17 cm with Performance Status 0 patients stratified by Eastern Cooperative Oncology Group performance [24]. The average setup errors are 0.13 ± 3.15 cm,0.37 ± 2.76 cm and 0.20 ± 5.73 cm in RL, AP and SI directions, respectively. And the maximum set-up margin in the present study was 0.67, 0.69 and 1.21 cm in RL, AP and SI directions calculated with van Herk recipe. These result was similar with the study by Wang L et al. [23], which assessed set-up margin using three-dimensional CBCT and report it to be 0.9–1.0 cm range. However, the study by Ueda Y et al. [16] assessed set-up margin by shift from MTP-4DCT at planning to MTP-cine at the treatment and reported it to be 0.52 cm in SI direction in 98% session’s calculated Stroom recipe (Table 2). The margin in study above is less than that in present study. We presume that their set-up, which used Body Fix double-vacuum system, resulted in their smaller set-up error.

The PTV margin also depends on the patient positioning techniques. Corradetti MN et al. [34] Stated that margins were 0.49 cm, 0.85 cm and 0.77 cm in RL, AP, and SI direction calculated by the van Herk recipe, respectively, which is smaller than the present result. We presume that their set-up, which used the full-body vacuum cushion system, resulted in their smaller inter-fractional error. Many other studies on SBRT have adopted 5 mm as the PTV margin using various advanced immobilized devices, such as Microtron, BodyFix. These devices can obviously reduce the PTV margin in the SBRT treatment for NSCLC. However, these advanced devices cause more time in the patients’ set-up procedure and bring more variations in the time spent on the treatment couch [17].

A total PTV margin combining tumor centroid, boundary changes and set-up displacement were 0.88, 0.98 and 2.68 cm in RL, AP and SI directions, respectively, which were maximum among all margin recipes. According to the comments of Table 5, the statistical assumption with van Herk’s recipe is minimum absorbed dose to CTV is 95% for 90% of patients. It’s requirement about minimum absorbed dose is high in realistic treatment plans, so the bigger margin size are needed to meet the requirement. This appoint is proved in my study, which shows that among all recipes, the maximum margin were calculated with the recipe by van Herk et al. And the requirement of Parker’s recipe about minimum absorbed dose is the same as one of van Herk’s, but the probability lever is not specified. And the results in my study shows that the margin calculated with Parker’s recipe is less than one with van Herk’s recipe.

**Table 5:** Summary of various published recommendations for margins around target volumes

Author	Recipe	Comments
Stroom et al. (1999a) [26]	2∑ + 0.7*σ*	95% absorbed dose to on average 99% of CTV tested in realistic plans.
Van Herk et al. (2000) [27]	2.5∑ + 0.7*σ*	Minimum absorbed dose to CTV is 95% for 90% of patients. Analytical solution for perfect conformation.
Parker et al. (2002) [28]	$\sum +\sqrt{\left({\sigma}^2+{\sum}^2\right)}$	95% minimum absorbed dose and 100% absorbed dose for 95% of volume. Probability levels not specified.
Snoke JJ (2007)	2.5∑ + *β*(*σ* ^2^ + *σ*_*P*_^2^)^1/2^ − *βσ*_*P*_	Minimum 95% of the prescribed dose for 90% of patients

Many studies stated that PTV margin was 0.5 cm in the axial plane and 1.0 cm in the cranial-caudal plane in all directions in the study on SBRT for NSCLC [20, 35–37]. The results were less than the one observed in SI direction of the present study. In conclusion, a 0.5 cm margin in the axial plane suffices to compensate the geometrical variety; nevertheless, a 1.0 cm margin in the SI plane is dose not compensated the geometrical variety in the present study, which the tumor located in lower lobe is adhered to chest wall and diaphragm.

## Conclusions

4DCT imaging is appropriate to account for the intra-fractional tumor centroid position, and boundary variations, and CBCT imaging is appropriate to account for the inter-fractional tumor set-up variation. We observed that the large tumor geometrical motion in lung tumor located in lower lobe and adhered to chest wall and diaphragm on SBRT for NSCLC and PTV margin could be as large as 0.88, 0.98 and 2.68 cm in RL, AP and SI directions, respectively. However, a conventional 1.0 cm margin in the SI plane dose not suffice to compensate the geometrical variety of the tumor adhere to chest wall and diaphragm.

## Abbreviations

4DCT: Four-dimensional computed tomography
CBCT: Cone-beam Computer Tomography
CTV: Clinical target volume
EPID: Electronic portal imaging device
GTV: Gross target volume
ITV: Internal clinical target volume
NSCLC: Non-small cell lung cancer
PTV: Planning target volume
SBRT: Stereotactic Body Radiotherapy
TP-3DCT: Treatment planning three-dimensional computed tomography
